# Human liver stem cell-derived extracellular vesicles enhance cancer stem cell sensitivity to tyrosine kinase inhibitors through Akt/mTOR/PTEN combined modulation

**DOI:** 10.18632/oncotarget.26319

**Published:** 2018-11-16

**Authors:** Valentina Fonsato, Michela De Lena, Stefania Tritta, Alessia Brossa, Ruggero Calvetti, Ciro Tetta, Giovanni Camussi, Benedetta Bussolati

**Affiliations:** ^1^ 2i3T, Società per la gestione dell'incubatore di imprese e per il trasferimento tecnologico, Scarl University of Torino, Torino, Italy; ^2^ Molecular Biotechnology Center, University of Torino, Torino, Italy; ^3^ Department of Molecular Biotechnology and Health Sciences, Torino, Italy; ^4^ Unicyte AG, Oberdorf, NW, Switzerland; ^5^ Department of Medical Sciences, University of Torino, Torino, Italy

**Keywords:** tumor stem cells, renal cell carcinoma, exosomes, Sunitinib, liver stem cell

## Abstract

It is well recognized that Cancer Stem Cells (CSCs) sustain the initiation, the maintenance and the recurrence of tumors. We previously reported that extracellular vesicles (EVs) derived from human liver stem cells (HLSCs) were able to limit tumor development. In this study, we evaluated whether EV derived from HLSCs could act in synergy with tyrosine kinase inhibitors (TKIs) on apoptosis of CSCs isolated from renal carcinomas. For this purpose, we administered to renal CSCs, HLSC-EVs and TKIs, as co-incubation or sequential administration. We found that HLSC-EVs in combination with Sunitinb or Sorafenib significantly increased renal CSCs apoptosis induced by low TKI dose. At variance, no synergistic effect was observed when bone marrow mesenchymal stem cell-derived EVs were used. In particular, renal CSCs chemosensitivity to TKIs was enhanced when HLSC-EVs were either co-administered with TKIs or added after, but not before. CSC apoptosis was also incremented at a percentage comparable to that of co-administration when TKIs were loaded in HLSC-EVs. By a mechanistic point of view, Akt/mTOR and Erk and Creb intracellular pathways, known to be pivotal in the induction of tumor growth and survival, appeared modulated as consequence of TKIs/HLSC-EVs co-administration. Together, our results indicate that the synergistic effect of HLSC-EVs with TKIs may increase the response to TKIs at low doses, providing a rational for their combined use in the treatment of renal carcinoma.

## INTRODUCTION

Renal cell carcinoma (RCC) is a common solid tumour associated to high mortality with frequent metastatic spread and high recurrence within 5 years [1]. Therapy for RCC is based on tumor surgical resection, as this tumor is highly resistant to common chemotherapeutic drugs [2, 3]. Over the last decade, a group of tyrosine kinase inhibitors (TKIs) was introduced in the clinical practice as first line treatment for metastatic RCC. Their anticancer activity is related to the inhibition of growth factor receptors overexpressed in renal cancer and responsible, at least in part, for tumor angiogenesis and cell proliferation [4]. Among TKIs, Sunitinib, and Sorafenib have conferred a good clinical outcome of patients in term of response rate, progression-free survival and overall survival [5]. Despite the clinical benefits, TKIs also display several adverse events such as hand-foot syndrome, mucosal inflammation, hypothyroidism and fatigue, together with hematological adverse events like anemia, leukopenia and thrombocytopenia [6]. Moreover, a major limit of TKI long-term anti-tumor effect is the development of resistance in the vast majority of cases [7].

It is now well recognized that the initiation, the maintenance and the recurrence of tumors is sustained by Cancer Stem Cells (CSCs), a small cell population with stem-like properties identified in several solid tumors [8–10]. In particular, in RCC, a CSC population has been identified by expression of surface endoglin (CD105) [11–15]. These renal CSC were shown to display several typical characteristics for cancer stem cells/tumor initiating cells, including clonogenicity, expression of stem cell markers and absence of differentiation markers, *in vitro* epithelial and endothelial differentiation ability, and generation of *in vivo* serially transplantable tumors with characteristics similar to the tumor of origin [11]. In consideration of the high drug resistance and tumor initiating capability of renal CSC, their targeting represents an important approach to eradicate RCC.

Cell-to-cell interaction is at least in part orchestrated by extracellular vesicles (EVs) that play a key role in cell communication by transferring mRNA, microRNA, lipids and proteins to target cells [16–18]. Tumor derived EVs were found to modulate tumor interstitial cell interaction and metastatic spread [19]. On the other hand, it was found that EVs derived from stem cells are able to reprogram tumor cells to a more benign phenotype, exerting their anti-tumor effect by blockade of proliferation and induction of apoptosis *in vitro* and by the regression of ectopic tumors *in vivo* [20, 21]. This anti-tumor activity was particularly evident for EVs derived from human liver stem cells (HLSC), a stromal cell population isolated from human adult liver that inhibited liver carcinomas as well as gliomas and lymphoblastomas [22].

In the present work, we investigated whether HLSC-EVs were able to exert an inhibitory effect *in vitro* on renal CSCs and to enhance the pro-apoptotic effect of TKIs, in different combination settings.

## RESULTS

### Co-administration of HLSC-EVs and TKIs increase apoptosis of rCSCs

**Figure 1:**
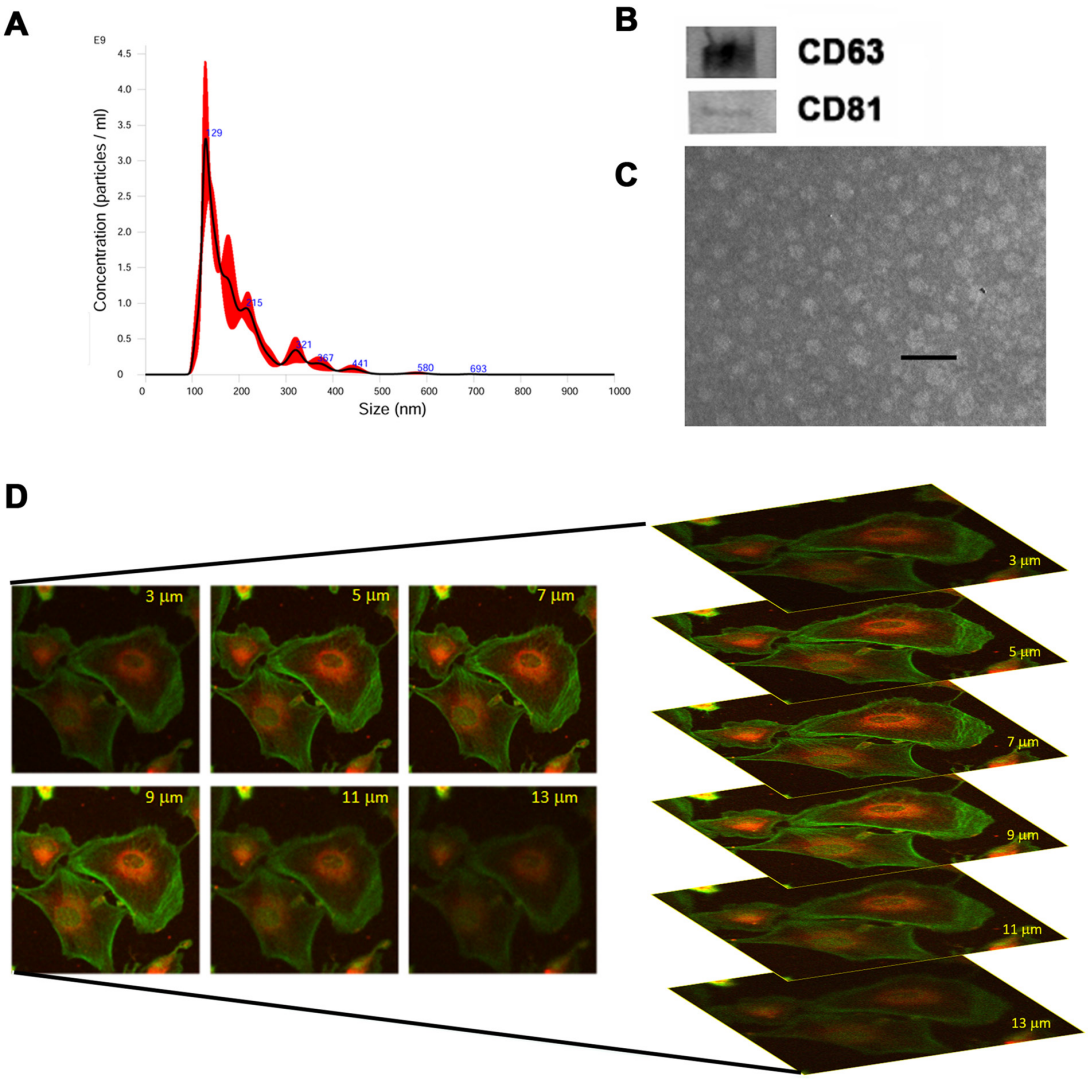
Characterization of EVs isolated from HLSCs **(A)** NanoSight size distribution graph showing the quantity and size of HLSC-EVs. **(B)** Representative Western blot analysis of CD63 and CD81 protein expression in HLSC-EVs. Data represent one of two experiments with similar results. **(C)** Representative electron microscopy of HLSC-EVs (scale bar = 100 nm). **(D)** Incorporation of DIL-labelled HLSC-EVs in G7 renal cells after 1 hour of incubation detected by confocal microscopy by z stack program (Original *magnification x400*).

Renal CSCs were isolated from renal carcinoma by magnetic cell sorting using selection for the CD105 surface antigen, and characterized as previously described [11]. Renal CSCs fulfilled the criteria of CSCs, including clonogenicity, expression of stem cell markers and generation of *in vivo* serially transplantable tumors (See Material and Methods and Supplementary Figure 1). To test the effect of stem cell derived EVs on chemosensitivity of renal CSCs, we isolated EVs from HLSC (HLSC-EVs) by ultracentrifugation. EVs were analyzed by NanoSight to quantify particle number and size (Figure 1A). Moreover, they were characterized by Western blot analysis for the expression of their characteristic markers CD63 and CD81 and by electron microscopy for their round cup-shape morphology (Figure 1B and 1C), as described [23]. When incubated with G7 renal CSCs, HLSC-EVs labelled with DIL dye were internalized by tumor cells after 1 hour of incubation at 37°C, as shown in Figure 1D. These characteristics are similar to those described for EVs derived by mesenchymal stromal cells (MSC-EVs) [23].

**Figure 2:**
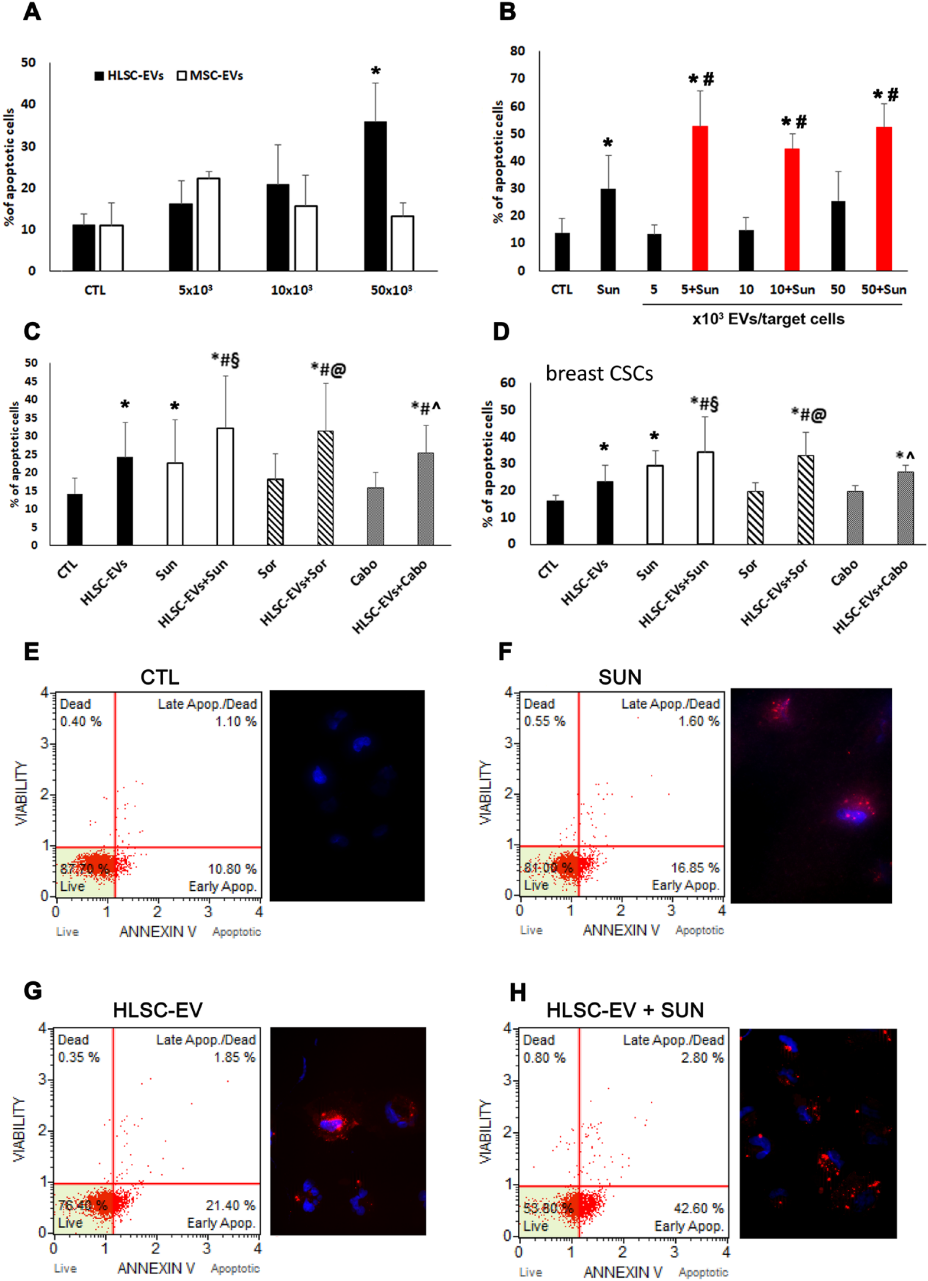
Apoptotic effect of HLSC-EVs on renal and breast CSCs **(A)** Analysis of apoptosis of G7 renal CSCs induced by different doses of HLSC-EVs (black column) and BM derived MSC-EVs (white column). G7 renal CSCs were stimulated for 48 hours with different doses of HLSC-EVs or MSC-EVs and Muse Annexin V & Dead Cell Assay evaluated apoptotic cells. **(B)** Apoptosis analysis of G7 renal CSCs stimulated with 1μM of Sunitinib alone and in combination with different doses of HLSC-EVs (5, 10, and 50 x 10^3^ EVs/target cells) for 48 hours. Data are mean ± SD of five different experiments (A and B): ^*^= *p* < 0.05 vs CTL cells; # = *p* < 0.05 vs Sunitinib. **(C)** Apoptosis analysis of G7 renal CSCs evaluated after 48 hours of treatment with HLSC-EVs (50 x 10^3^ EVs/target cells), Sunitinib (1μM), Sorafenib (5μM) and Cabozantinib (2μM) alone or in combination (HLSC-EVs+Sun, HLSC-EVs+Sor, HLSC-EVs+Cabo). **(D)** Apoptosis analysis of C10 breast CSCs stimulated for 48 hours with HLSC-EVs (50 x 10^3^ EVs/target cells), Sunitinib (1μM), Sorafenib (5μM) and Cabozantinib (2μM) alone or in combination (HLSC-EVs+Sun, HLSC-EVs+Sor, HLSC-EVs+Cabo). Data are mean ± SD of three different experiments (C and D): ^*^= *p* < 0.05 vs CTL; # = *p* < 0.05 vs HLSC-EVs; § = *p* < 0.05 vs Sunitinib; @ = *p* < 0.05 vs Sorafenib; ^ = *p* < 0.05 vs Cabozantinib. **(E-H)** Representative cytofluorimetric analysis of dead and apoptotic cells as evaluated by Muse™ Annexin V and Dead Cell Assay and immunofluorescence images of Annexin V staining of renal CSCs when treated with HLSC-EV, Sunitinib alone or their combination. Nuclei are stained with Hoechst 33342 (blu). (Original *magnification x400*).

The effect of EVs on CSC apoptosis was evaluated after treatment of different doses (5, 10, 50 × 10^3^ EV/target cell) of HLSC-EVs and MSC-EVs. As shown in Figure 2A, HLSC-EVs, but not MSC-EVs, exerted a dose dependent pro-apoptotic effect on G7 renal CSC that was statistically significant at the dose of 50 × 10^3^ EV/target cell (Figure 2A). In order to evaluate a possible combinatory effect of EVs with Sunitinib, we first performed experiments to evaluate the TKI dose-response on renal CSCs (Supplementary Figure 2A). We selected the dose of Sunitinib showing the minimal pro-apoptotic effect (1 μM; Supplementary Figure 2). The percentage of renal CSC apoptosis was significantly increased when HLSC-EVs were co-administered with 1 μM Sunitinib compared to Sunitinib alone as well as to HLSC-EVs alone, as evaluated by Annexin V Dead Cell Assay and by Annexin V immunofluorescence staining (Figure 2B, 2E-2H). In particular, an increment of about 40% of apoptosis was observed already at the low non-apoptotic dose of 5 × 10^3^ HLSC-EVs/target cell in combination with 1 μM Sunitinib (Figure 2B).

In order to test whether the apoptotic effect was specific for Sunitinib or common to other TKIs used for the treatment of metastatic RCC, we also tested Sorafenib and Cabozantinib alone and in combination with HLSC-EVs. The minimal apoptotic dose of 5 μM was used for Sorafenib (Supplementary Figure 2B) and of 2 μM for Cabozantinib (Supplementary Figure 2C). Figure 2 shows that both the Sorafenib/HLSC-EVs and Cabozantinib/HLSC-EVs co-administration induced an enhancement of apoptotic cells, with a similar effect to the Sunitinib/HLSC-EVs co-administration. Moreover, this increment was significant not only in respect to control cells, but also to cells stimulated with HLSC-EVs or TKIs alone (Figure 2C).

Finally, to evaluate whether the observed effect on chemosensitivity was specific for renal CSCs or could be shared by CSCs of different origin, the effect of HLSC-EVs and Sunitinib/Sorafenib/Cabozantinib co-administration was evaluated also on C10 breast CSCs. The results showed a significant apoptotic increment similar to that observed for renal CSCs (Figure 2D).

### Pro-apoptotic effect of HLSC-EVs loaded with TKIs

**Figure 3:**
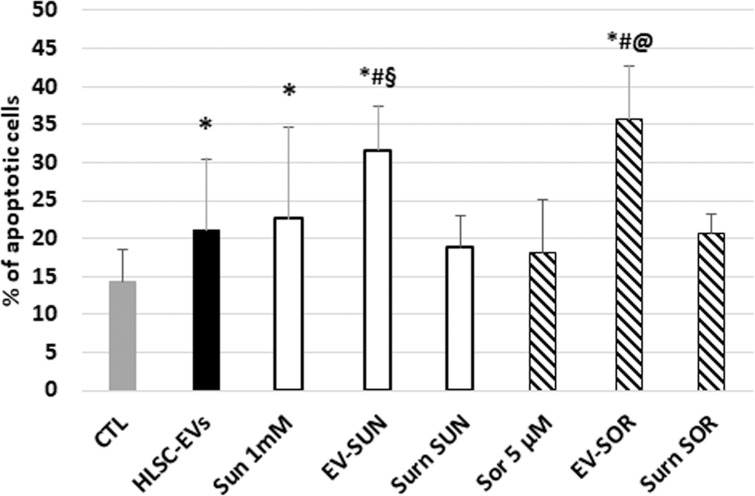
Effect of loading of HLSC-EVs with TKIs on G7 renal CSCs apoptosis Apoptosis analysis of G7 renal CSCs treated with Sunitinib, Sorafenib, or with HLSC-EVs loaded with Sunitinib (EV-SUN) or with Sorafenib (EV-SOR). The supernatant (surn-SUN and surn-SOR) after ultracentrifugation was recovered used as negative control. Data are mean ± SD of three different experiments: ^*^= *p* < 0.05 vs CTL; # = *p* < 0.05 vs HLSC-EVs; § = *p* < 0.05 vs Sunitinib; @ = *p* < 0.05 vs Sorafenib.

To assess whether the pro-apoptotic effect of HLSC-EV/TKI co-administration could be further increased using TKI-loaded EVs, we generated HLSC-EVs loaded with Sunitinib or Sorafenib. As these TKIs are lipophilic [24], we simply co-incubated EVs with TKIs for 15 minutes followed by ultracentrifugation to wash out the unbound drugs. The EVs obtained were called EV-SUN or EV-SOR to indicate EVs loaded with Sunitinib or Sorafenib respectively. The effective drug loading within EVs was determined by spectrum analysis (see Mat and Methods) and the amount of EVs was calculated in order to reach the same TKI concentration used in experiments above. As shown in Figure 3, the pro-apoptotic effect of HLSC-EVs loaded with TKIs was comparable to that of HLSC-EVs and TKIs co-administration (Figure 3), suggesting that that drug-loaded EVs could be an alternative approach for drug delivery.

### HLSC-EVs co-administered with TKIs inhibited the Akt/mTOR/PTEN pathway

**Figure 4:**
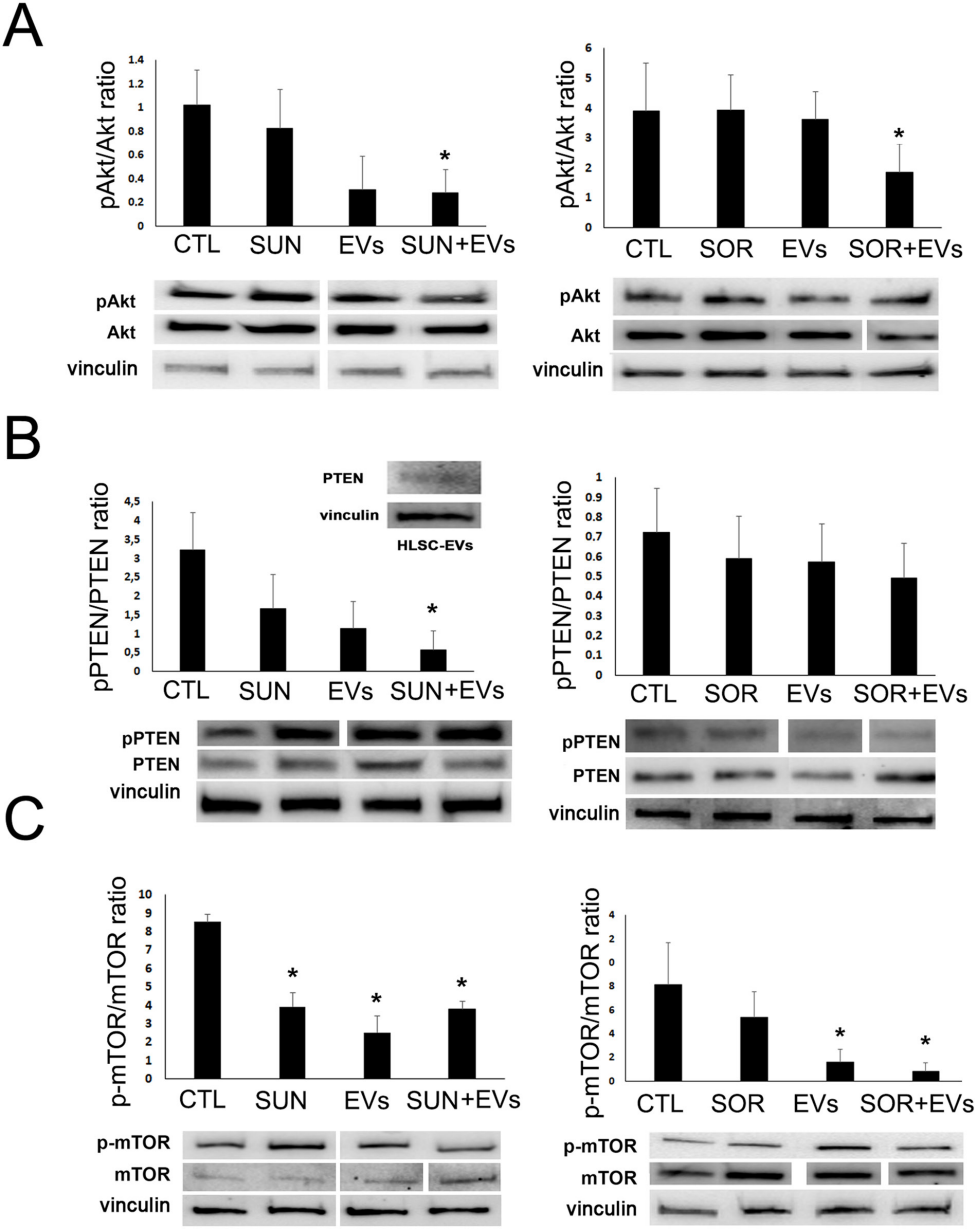
HLSC-EVs/TKIs co-administration decreased phospho-proteins in renal CSCs Western blot micrograph and densitometric analysis of pAkt/Akt **(A)**, pPTEN/PTEN **(B)** and p-mTOR/mTOR **(C)** ratio on G7 renal CSCs stimulated for 3 hours with HLSC-EVs (50 x 10^3^ EVs/target cells), Sunitinib (1μM), Sorafenib (5μM) alone or in combination (HLSC-EVs+Sun, HLSC-EVs+Sor). Data, shown as arbitrary units, were representative of three different experiments and were normalized to vinculin expression. ^*^= p < 0.001 vs CTL. *Inset:* Western blot micrograph of PTEN expression by HLSC-EVs.

**Figure 5:**
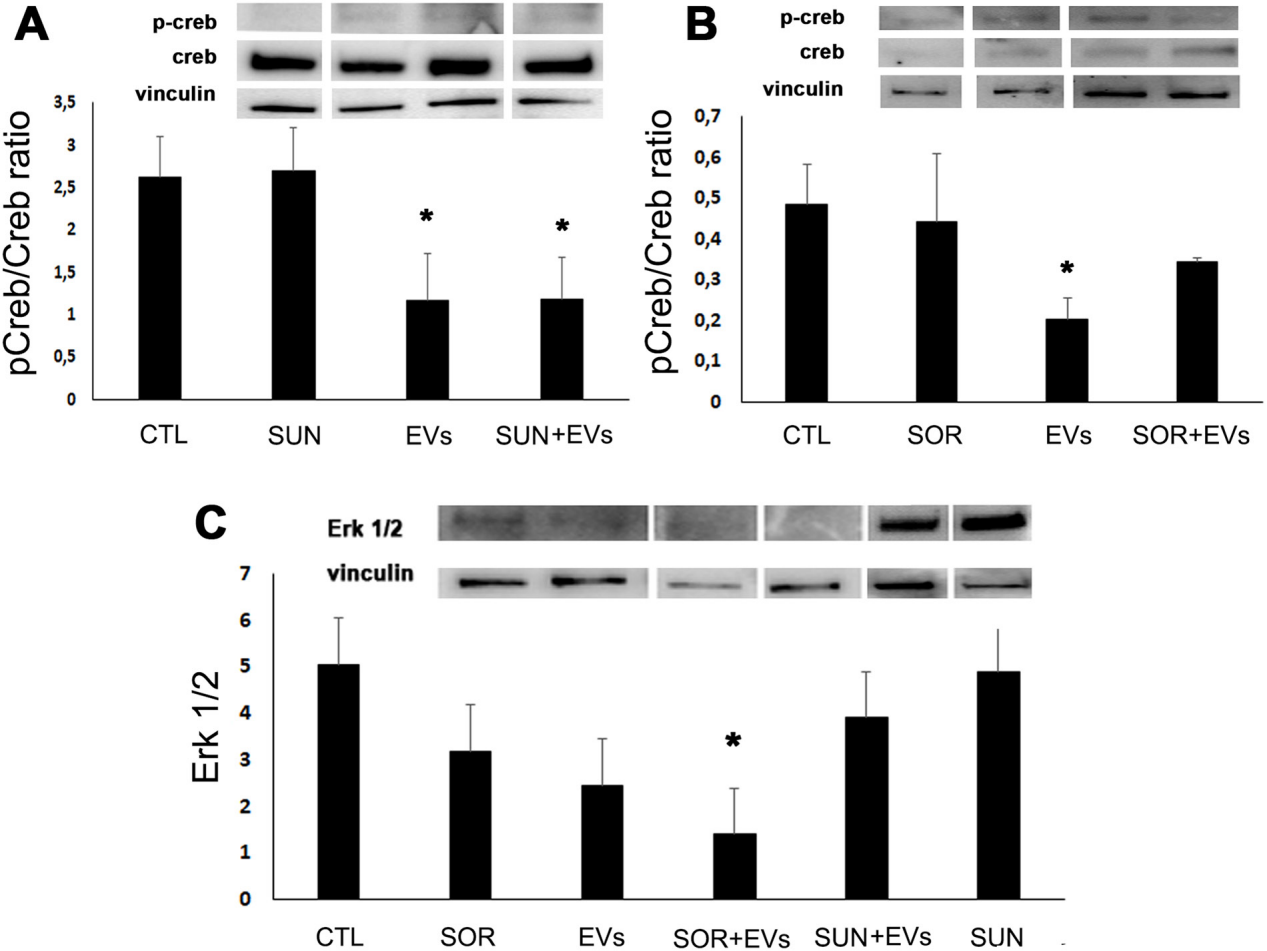
HLSC-EVs inhibited pCreb/Creb ratio and, when combined with Sorafenib, Erk 1/2 Western blot micrograph and densitometric analysis of pCreb/Creb ratio **(A** and **B)** and of Erk 1/2 **(C)** on G7 renal CSCs stimulated for 3 hours with HLSC-EVs (50 x 10^3^ EVs/target cells), Sunitinib (1μM), Sorafenib (5μM) alone or in combination (HLSC-EVs+Sun, HLSC-EVs+Sor). Data, shown as arbitrary units, were representative of three different experiments and were normalized to vinculin expression. ^*^= p < 0.001 vs CTL.

In order to understand the mechanism underlying the pro-apoptotic effect exerted by co-administration of TKIs and HLSC-EVs, we performed a Bio-Plex Pro cell signaling assay for the detection of intracellular phosphoproteins (not shown). The results were then validated by Western blot analysis. As shown in Figure 4, we found that TKIs and HLSC-EVs co-administration induced a synergistic effect in respect to TKIs or EVs alone on specific pathways. In particular, the co-administration of Sunitinib and HLSC-EVs was able to reduce Akt activity and enhance the oncosuppressor PTEN trough decrease of its phosphorylated form in respect to treatments alone (Figure 4A and 4B). The Akt/PTEN pathways was inhibited also by co-administration of Sorafenib and HLSC-EVs, even if the reduction of pPTEN/PTEN ratio did not reach significance (Figure 4A and 4B). In addition, we found that the PTEN protein was directly expressed by HLSC-EVs (Figure 4B, inset). Moreover, HLSC-EVs alone significantly reduced the active phosphorylated form of mTOR (Figure 4C) and the activation of the Creb transcription factor (Figure 5A and 5B).

Finally, the Sorafenib/HLSC-EV co-administration induced a significant decrease of Erk 1/2 (Figure 5C) that was specific for this TKI, as no synergistic effect was observed with Sunitinib/HLSC-EV (Figure 5C).

### EV post-incubation but not pre-incubation increase chemosensitivity

**Figure 6:**
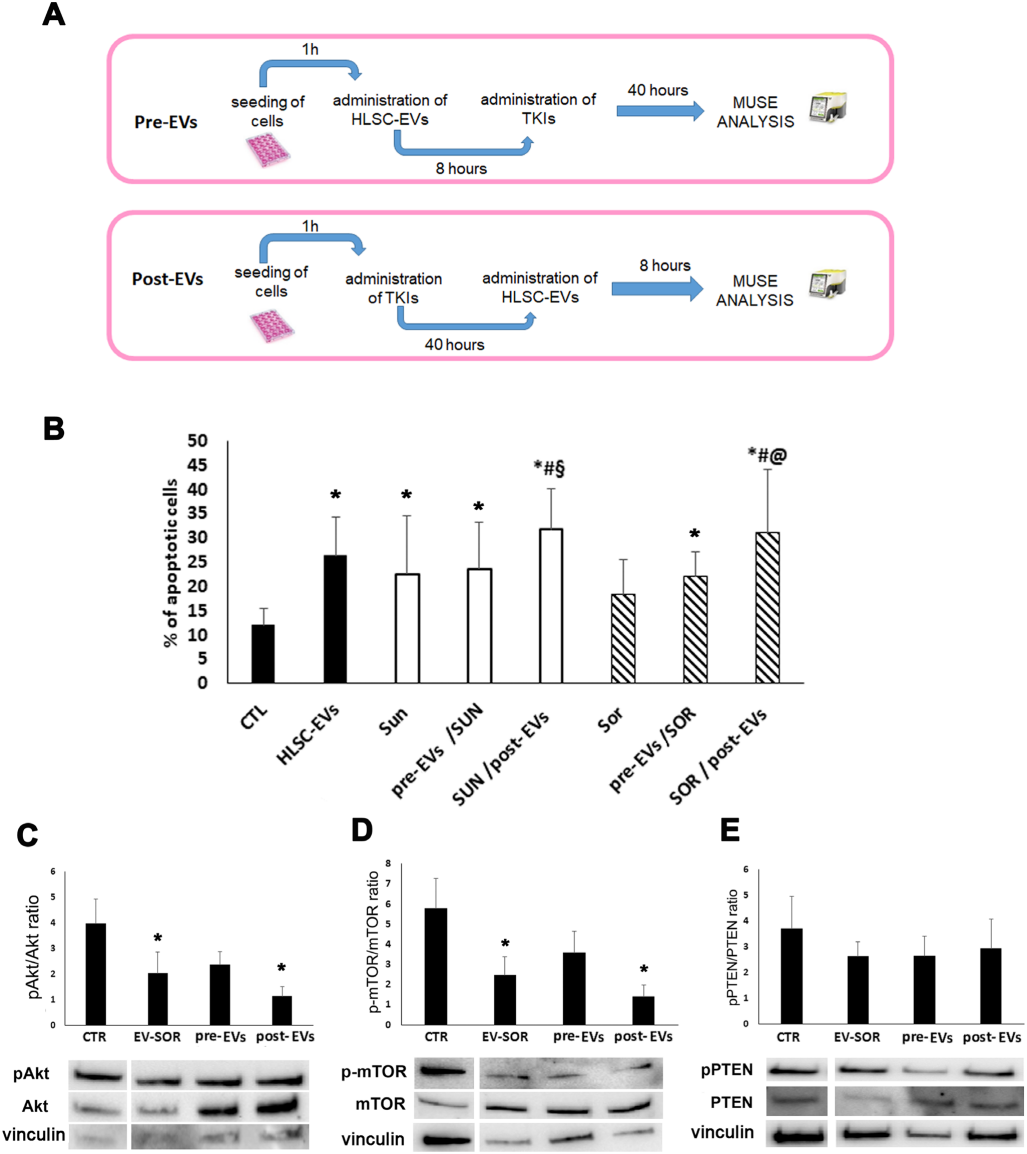
Effect of HLSC-EVs and TKIs sequential administration on G7 renal CSCs apoptosis **(A)** Schematic representation of the sequential administration experiment. Pre-EVs: G7 renal CSCs were incubated with HLSC-EVs for 8 hours and then stimulated with Sunitinib (1 μM) or Sorafenib (5 μM) for additional 40 hours. Post-EVs: G7 renal CSCs were stimulated with Sunitinib or Sorafenib for 40 hours and then stimulated with HLSC-EVs for additional 8 hours for a total of 48 hours of incubation. **(B)** Apoptosis analysis of G7 renal CSCs after 48 hours of sequential treatment. Data are mean ± SD of three different experiments: ^*^= *p* < 0.05 vs CTL; # = *p* < 0.05 vs HLSC-EVs; § = *p* < 0.05 vs Sunitinib; @ = *p* < 0.05 vs Sorafenib. **(C-E)** Western blot micrograph and densitometric analysis of pAkt/Akt (C), p-mTOR/mTOR (D) and pPTEN/PTEN (E) ratioon CSCs after sequential administration of HLSC-EVs (3 hours) and Sorafenib (40 hours). Data, shown as arbitrary units, were representative of three different experiments and were normalized to vinculin expression: ^*^= p < 0.001 vs CTL.

We also performed experiments of sequential administration of HLSC-EVs and TKIs to better dissect the synergistic effect of EVs on TKI induced apoptosis. We first incubated renal CSCs with HLSC-EVs for 8 hours and then stimulated with Sunitinib (1 μM) or Sorafenib (5 μM) for additional 40 hours, to reach 48 hour incubation used in co-administration experiments (Figure 6A). On the other hand, we incubated renal CSCs with Sunitinib or Sorafenib for 40 hours and then stimulated with HLSC-EVs for additional 8 hours (Figure 6A). The results indicated that only the post- incubation and not the pre-incubation of HLSC-EVs enhanced the chemosensitivity of renal CSCs to TKIs (Figure 6B). This further suggests that the effect of HLSC-EVs/TKI combination on chemosensitivity was due to an EV-dependent enhancement of TKI induced mechanisms and not to epigenetic changes induced by EV leading to increased TKI sensitivity. This notion was supported by the analysis of the p-AKT/p-mTOR and PTEN pathways of renal CSCs pre- or post-treated with EVs. Indeed, a significant additive effect of EVs in reducing the phosphorylation of Akt/mTOR pathway was observed only when EVs were administered after Sorafenib (Figure 6C-6E).

**Figure 7:**
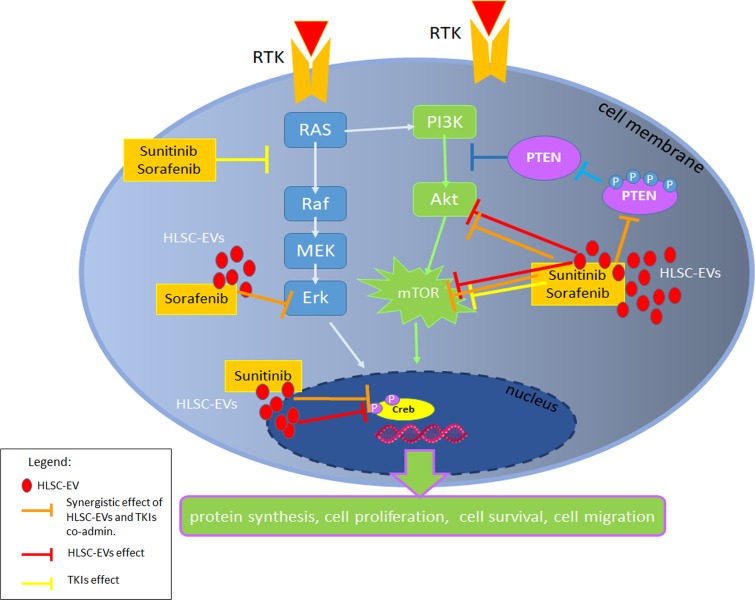
Survival pathways modulated by HLSC-EVs/TKIs co-administration Schematic representation of the inhibitory effect of HLSC-EVs and of TKIs alone or in combination on PI3K pathway and Erk. In particular, the co-administration of HLSC-EVs/TKIs exerted a synergistic effect (orange bars) on the inhibition of Akt and mTOR pathways and on pPTEN. HLSC-EVs alone (red bars) showed an inhibitory effect on Akt, mTOR pathways and on transcription factor Creb. Sunitinib or Sorafenib alone (yellow bars) inhibit RAS and mTOR pathways.

Altogether, these results, summarized in Figure 7, indicate that EVs synergize with TKI in inducing apoptosis by interfering with renal CSC survival pathways.

## DISCUSSION

In this study, we demonstrate a specific synergistic effect of HLSC-EVs with TKIs in inducing apoptosis of renal and breast CSCs. In particular, renal CSCs chemosensitivity to TKIs was enhanced when HLSC-EVs were co-administered or sequentially added after TKIs. The observed effect can be ascribed to a combined modulation of the Akt/mTOR and Erk survival pathways by HLSC-EV/TKI co-administration.

Renal cell carcinoma is considered one of the fastest increasing cancers over the next 20 years. Every year, 63,920 [25] and 115,200 [26] estimated new cases of kidney cancer in the United States and in Europe, respectively, are registered with estimate death cases of 13,860 and 49,000. Nowadays, a group of TKIs, which comprises Sunitinb and Sorafenib, represents the first line treatment for metastatic RCC [27]. Unfortunately, the TKI-based therapy is often correlated with several adverse effects and with development of resistance [28], highlighting the importance to improve the benefit of the TKIs limiting the related complications.

Several groups showed an anti-tumor activity by bone marrow MSC-EVs and placental stem cell-derived EVs [20, 29–32]. We here showed that, as reported for other tumor cell types, [22], HLSC-derived EVs, induced a significant degree of apoptosis in renal CSCs. No effect of MSC-EVs was observed in this model. Interestingly, we found that HLSC-EVs directly modulated CSC intracellular pathways (Akt, mTOR and Creb), in analogy with the reported effect of MSC-EVs on the direct modulation of protein FAK phosphorylation in myeloma cells [33]. Moreover HLSC-EVs contained PTEN that could be possibly transferred to the target CSCs. Indeed, transfer of signalling molecules packaged into exosomes [34] was also reported for tumor exosomes.

We also found that when HLSC-EVs were combined with Sunitinib at a minimally affecting dose (1 μM), the percentage of renal CSC apoptosis was significantly increased compared to Sunitinib alone as well as to HLSC-EVs alone. The increase was of particular evidence (about 40% increase of apoptosis) at the lowest non-apoptotic dose of HLSC-EVs when combined with Sunitinib. These results appear of interest for improving the effect of TKIs, as they were common to different drugs and to CSC from other tumors. In fact, HLSC-EVs showed a synergic effect when co-administered also with Sorafenib and Cabozantinib. Moreover, the combination of HLSC-EVs with different TKIs was effective not only on renal CSCs, but also on breast CSCs, supporting the hypothesis that the combination of EVs with anti-cancer drugs could be useful in clinical application to treat tumors of numerous origin. *in vivo* experiments of synergistic administration would be required to confirm this effect. Indeed, EVs present several advantages as possible therapeutic tools [35, 36] as they possess a high biocompatibility and can easily diffuse through tissue due to their small size.

In the attempt to identify possible strategies for a clinical administration, we performed experiments of sequential administration that indicated a benefit when EVs were added after TKIs. This may suggest that EVs may act of CSCs by enhancing TKI induced mechanisms rather than by inducing epigenetic changes and leading to increased TKI sensitivity. This appears a different mechanism in respect to that observed for cancer treatment with normal prostate cell-derived EVs that reverted paclitaxel resistance [37]. An alternative strategy for EV and TKI administration can be envisaged in the direct EV loading with TKIs. HLSC-EVs loaded with Sunitinib and Sorafenib enhanced rCSC apoptosis in a similar manner to that of co-administration. These results confirmed that EVs may represent an alternative therapeutic drug delivery carrier not only for their specific homing ability but also for their higher loading ability to hydrophobic chemical drug in respect to liposomes [38, 39].

The mechanism responsible for the anticancer effect of TKIs mainly involve a negative regulation of the survival and proliferative intracellular pathways related to target growth factor receptor activation. The Phosphoinositide 3-kinase (PI3K)/AKT/mTOR and Ras/Mek/Erk have been demonstrated as the most critical cell signalling pathways in carcinogenesis and display a cross talk between them [40]. In particular, PI3K/Akt/mTOR pathway modulates cell proliferation, survival, motility, invasion allowing cancer initiation and progression [41]. In addition, the MAPK pathway also regulates cell proliferation, growth, and survival [42]. We here found that HLSC-EVs alone modulated the classical targets of TKIs, and that the combination of EVs and TKIs further increased the observed effects. In fact, HLSC-EVs alone displayed a negative effect on mTOR. The co-administration of HLSC-EVs with both Sunitinib and Sorafenib induced a reduction of phospho-Akt and phospho-mTOR, blocking their pro-survival activity. Moreover, the HLSC-EV/TKI treatment modulated the onco-suppressor PTEN, which in turn controls the PI3K/Akt signalling pathway [43]. In particular, HLSC-EVs/TKIs treatment induced a reduction of phospho-PTEN, the inhibitory form of PTEN, with resulting increase of its level and activity. This effect may be relevant for the control of CSCs, as the increase of p-PTEN/PTEN ratio sustains the acquisition of malignant phenotypes for many types of cancer [44]. Furthermore, the combination treatment of HLSC-EVs with Sorafenib induce an inhibition of Erk.

The effect of TKI/EV combination on the PI3K/AKT/mTOR and Ras/MEK/ERK is confirmed by a significant decrease of the levels of pCreb, the transcription factor involved in their signalling to promote tumor growth, apoptotic resistance, angiogenesis, and distant metastasis [45]. In particular, EVs alone showed a significant decrease of pCreb that was further reduced when rCSCs were stimulated with a combination treatment of HLSC-EVs/Sunitinib.

Together, our results indicated that HLSC-EVs and TKIs have a synergistic anti-tumor effect on renal CSCs inducing an enhancement of apoptosis by a combined effect on Akt/mTOR, Erk and Creb intracellular pathways, known to be pivotal in the induction of tumor growth and survival. The synergistic effect of HLSC-EVs with TKIs at low doses may increase the response to TKIs on CSCs and provide a rational for a combined use of these products in the treatment of renal carcinoma.

## MATERIALS AND METHODS

### Cancer stem cells isolation and culture

Renal CSCs were isolated and characterized as previously described [11, 46]. Briefly, CSCs were obtained from specimens of renal cell carcinomas from patients undergoing radical nephrectomy according to the Ethics Committee of the S. Giovanni Battista Hospital of Torino, Italy (168/2014). Cells were isolated, using anti-CD105 Ab coupled to magnetic beads, by magnetic cell sorting using the magnetic-activated cell sorting (MACS) system (Miltenyi Biotec, Auburn, CA, USA) from renal carcinomas (histological types: 3 clear-cell type and 2 undifferentiated carcinomas). Briefly, cells were labelled with the anti-CD105 mAb for 20 min, washed twice and resuspended in MACS buffer (PBS without Ca2 and Mg2, supplemented with 1% BSA and 5 mM EDTA) at a concentration of 2×10^7^ cells. After washings, cells were separated on a magnetic stainless steel wool column (Miltenyi Biotec), according to the manufacturer's recommendations. Magnetically sorted CD105^+^ CSCs were cultured in the presence of the expansion medium, consisting of DMEM LG (Invitrogen), with insulin-transferrin-selenium, 10^−9^ M dexamethasone, 100 U penicillin, 1000 U streptomycin, 10 ng/ml EGF (all from Sigma-Aldrich) and 5% fetal calf serum (FCS) (Sigma-Aldrich). For cell cloning, single cells were seeded in 96-well plates in presence of the expansion medium. A G7 CD105^+^ clonal renal cell carcinoma stem cell line was selected and used for all the experiments. Immunophenotypic analysis showed the positivity for CD105, expression of the mesenchymal stem cell marker CD73, SSEA4 and the absence of CD133 and CD24 and EPCAM (Supplementary Figure 1A). When cultured in non-adhesive culture conditions, G7 renal CSCs were able to growth and form spheres that could be propagated for several passages (Supplementary Figure 1B). *In vivo*, when implanted subcutaneously in SCID mice, G7 renal CSC were able to generate tumors using a low number of cells, such as 1×10^3^ and 1×10^2^ cells (Supplementary Figure 1C). Moreover, CD105^+^ cells isolated from tumors were re-injected in SCID mice and gave raise to secondary and tertiary tumors (Supplementary Figure 1D), confirming their identity as tumor-initiating cells.

Breast CSCs were isolated from breast lobular-infiltrating carcinoma obtained as previously described [47]. Briefly, tumour specimen was finely minced with scissors and digested by incubation for 1 h at 37°C in DMEM containing collagenase II (Sigma Chemical Company, St. Louis, MO, USA). After washings in medium plus 10% FCS (GIBCO, Grand Island, NY, USA), the cell suspension was forced through a graded series of meshes to separate the cell components from stroma and aggregates and, finally, through a 40-μm pore filter (Becton Dickinson, San Jose, CA, USA). Single cells were plated at 1000 cells/ml in serum-free DMEM-F12 (Cambrex BioScience, Venviers, Belgium), supplemented with 10 ng/ml basic fibroblast growth factor (bFGF), 20 ng/ml epidermal growth factor (EGF), 5 (μg/ml insulin and 0.4% bovine serum albumin (all from Sigma), as described by Ponti et al. [48]. For cell cloning, single cells were seeded in 96-well plates in presence of the expansion medium. A C10 clonal breast cell carcinoma stem cell line (C10 breast CSCs) was selected and used for all the experiments.

### Human mesenchymal and liver stromal stem cells

HLSC were isolated from human cryopreserved normal hepatocytes obtained from Lonza (Basel, Switzerland, https://www.lonza.com/), characterized and cultured as previously described [49, 50]. Human hepatocytes were plated in the presence of alfa minimum essential medium/endothelial cell basal medium (expansion media: ɑMEM/EBM in the ratio 3:1, Lonza), supplemented with antibiotics (100 U penicillin and 1,000U streptomycin; both from Sigma, St. Louis) and 10% Foetal Calf Serum (FCS, Sigma). After 2 week HLSC colonies were evident were expanded.

Bone marrow-derived mesenchymal stem cells (MSCs) were obtained from Lonza and cultured and characterized as previously described [51]. MSCs were used up to the sixth passage of culture. All of the cell preparations used were positive for the typical MSC markers (CD105, CD29, CD73, CD44, and CD90 (not shown)).

### EVs isolation

The supernatant of HLSCs or MSCs was recovered and centrifuged for 20 min at 3000 g to remove cell debris and apoptotic bodies. An ultracentrifugation at 100,000 g for 2 hours at 4°C (Beckman Coulter Optima L-90 K, Fullerton, CA, USA) has followed the previous one. Both HLSC-EVs and MSC-EVs were resuspended in RPMI supplemented with 1% dimethyl sulfoxide (DMSO) and frozen at −80°C for later use. Concentration and size distribution of EVs were determined by the Nanosight LM10 system (NanoSight, Wiltshire, UK). Briefly, EV preparations were diluted (1:200) in sterile saline solution and analyzed by the Nanoparticle Analysis System using the NTA 1.4 Analytical Software (Figure 1A) [52]. To evaluate the internalization of EVs in G7 renal CSCs by fluorescent microscopy, EVs were labelled with 1 μM Dil dye (Thermo Fisher Scientific, Waltham, MA, USA) as described previously [52]. Briefly, purified EVs were resuspended in PBS supplemented with 1 μM Dil dye and ultracentrifuged at 100,000 g for 1 h at 4°C. Following labelling, the EVs were washed with PBS by ultracentrifugation as mentioned above. The pellet obtained was then resuspended in RPMI with 1% DMSO and frozen for subsequent studies.

### Co-administration of HLSC-EVs and TKIs increase apoptosis of rCSCs

Renal CSCs were incubated with EVs derived from MSC or HLSCs.

For loading experiments, EVs were loaded with 10 μM of Sunitinib or 50 μM of Sorafenib by incubating together for 15 minutes at 37°C and then ultracentrifuged at 100,000 g for 1 h at 4°C to remove the unloaded drug. EVs were resuspended in RPMI with 1% DMSO and named EV-SUN those loaded with Sunitinib or EV-SOR those loaded with Sorafenib. The supernatant (surn-SUN and surn-SOR) was recovered and used in the experiments as negative control. The dose of Sunitinib and Sorafenib used was chosen on the base of preliminary experiments showing 8-10% of drug incorporation, as described [53]. Spectrum analysis was used to evaluate the effective drug loading within EVs and it revealed the presence of 1.8 μM Sunitinib and 10 μM for Sorafenib in EV-SUN or EV-SOR (not shown).

### Apoptosis assay

Apoptosis was evaluated by Muse™ Annexin V and Dead Cell Assay (Millipore, Merck KGaA, Darmstadt, Germany) according to manufacturer's instructions. The assay is based on the detection of phosphatidylserine (PS) on the surface of apoptotic cells, using fluorescently labeled Annexin V in combination with the dead cell marker, 7-AAD. Briefly, G7 renal CSCs or C10 breast CSCs were seeded at the concentration of 2×10^4^ cells/well and, after cell attachment, were stimulated with HLSC-EVs or Sunitinib or Sorafenib alone or in combination and cultured for 48 hours. At the end of the incubation period, the supernatant containing dead cells and cells were recovered, incubated for 20 minutes with Annexin V/7-AAD reagent and read at Muse. The results were showed as the percentage of total apoptotic cells.

### Immunofluorescence

For immunofluorescence experiments, renal CSCs were seeded at the concentration of 2×10^4^ cells/well and, after cell attachment, were stimulated with HLSC-EVs (50×10^3^/target cells) or Sunitinib alone or in combination and cultured for 48 hours. At the end of the incubation time, cells were stained with Annexin V/7-AAD reagent for 20 minutes at room temperature in the dark. Then, cells were washed and paraformaldehyde fixed. Nuclei were stained with Hoechst 33342. Microscopy analysis was performed using a LSM5 Pascal confocal microscope (Carl Zeiss International).

### Phospho-protein array

Intracellular phosphoproteins were evaluated in the lysates of renal G7 CSCs by the magnetic bead-based immunoassays Bio-Plex Pro cell-signaling assay according to manufacturer's instruction (BIoRad, Hercules, California, US). Briefly, cells were treated or not with Sunitinib (1 μM) or Sorafenib (5 μM) or HLSC-EVs (50 × 10^3^ EV/target cell) or with the co-administration of HLSC-EVs/Sunitinib or HLSC-EVs/Sorafenib for three hours. Then, cells were lysed and lysates were incubated with capture antibodies coupled to the beads. Coupled beads react with the sample containing the analyte of interest. After a series of washes to remove unbound protein, a biotinylated detection antibody was added to create a sandwich complex. The final detection complex was formed with the addition of streptavidin-phycoerythrin (SA-PE) conjugate and submitted to Bio-Plex system with Bio-Plex Manager software analysis.

### Western blot analysis

G7 renal CSCs were stimulated for 3 hours with HLSC-EVs alone or in combination with Sunitinib or Sorafenib. In the sequential administration experiments, G7 renal CSCs were stimulated for 3 hours with HLSC-EVs before or after treatment with Sorafenib (5 μM, 40 hour stimulation). At the end of incubation time, cells were lysed in RIPA buffer supplemented with protease and phosphatase inhibitor cocktail and PMSF (Sigma-Aldrich). Aliquots of the cell lysates containing 30 μg proteins form cells or 10 μg from EVs, as determined by the Pierce™ BCA Protein method (Thermo Scientific, Rockford, IL, USA), were run on 4-20% SDS-PAGE under reducing conditions and blotted onto PVDF membrane filters using the iBLOT system (Life Technologies). The membranes were blocked in Tris-buffered saline-Tween (TBS-T; 25 mM Tris, pH 8.0, 150 mM NaCl, and 0.05% Tween-20) containing 5% (w/v) non-fat dried milk for 1 h. After blocking, membranes were probed overnight with primary antibody. Anti-vinculin (Santa Cruz Biotechnology), anti-AKT or anti p-AKT (Ser473), anti-PTEN or anti-pPTEN, anti-mTOR or anti-p-mTOR, anti-Creb or anti-pCreb and anti-Erk 1/2 (all from Cell Signalling) primary Abs were used. After extensive washings with TBS-T, the blots were incubated with appropriate peroxidase conjugated secondary antibodies for 1 h at room temperature. Goat anti-Rabbit IgG and goat anti-mouse IgI HRP conjugated secondary antibodies (Thermo Scientific, Rockford, IL, USA) were used. Following incubation, the membranes were washed extensively with TBS-T, probed with ClarityTM Western ECL substrate (Bio-rad, CA, USA), and detected by the Chemidoc system (Bio-rad, CA, USA).

### Statistical analysis

All data of different experiments were expressed as mean ± SD. Statistical analysis was performed by ANOVA with Newmann Keuls' multicomparison post hoc test. Two-tailed *p* value ≤ 0.05 was considered statistically significant.

## SUPPLEMENTARY MATERIALS FIGURES



## Abbreviations

RCC: Renal Cell Carcinoma
CSC: Cancer Stem Cells
HLSC: human liver stem cells
EV: extracellular vesicles
TKI: tyrosine kinase inhibitor
SUN: sunitinib
SOR: sorafenib
MSC: mesenchymal stem cells
RTK: tyrosine kinase receptor
PTEN: phosphatase and tensin homolog
mTOR: mammalian target of rapamycin
Akt: protein chinase B or PKB
PI3K: phosphoinisitide 3-kinase
Erk: extracellular signal-regulated kinases
