# Supplementary material for: Human liver stem cell-derived extracellular vesicles enhance cancer stem cell sensitivity to tyrosine kinase inhibitors through Akt/mTOR/PTEN combined modulation
Source: Oncotarget. 2018 Nov 16;9(90):36151–65. doi: 10.18632/oncotarget.26319 (PMC6281417; doi:10.18632/oncotarget.26319)
Supplement: Supplementary file 1 [file oncotarget-09-36151-s001.pdf]

Human liver stem cell-derived extracellular vesicles enhance cancer stem cell sensitivity to tyrosine kinase inhibitors through Akt/mTOR/PTEN combined modulation

SUPPLEMENTARY MATERIALS

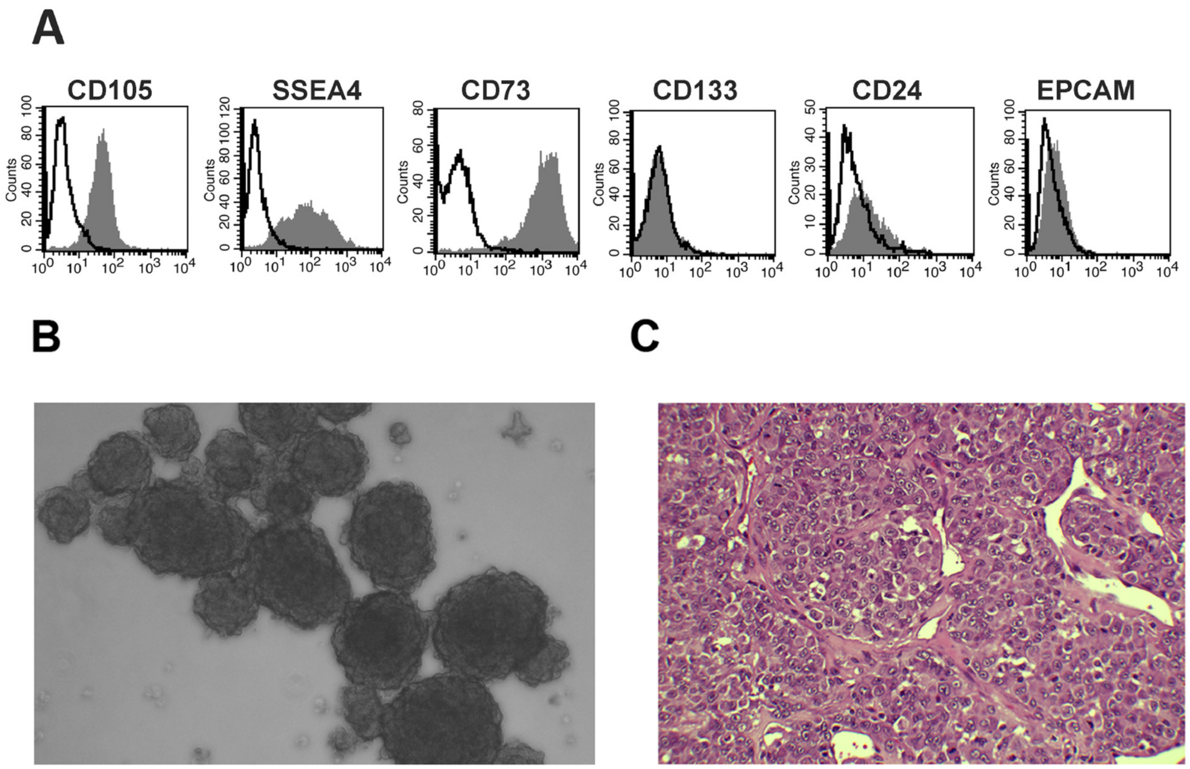

D

Generation of serially transplantable tumors from renal CSC G7 clone

G7 CSCs	passage		
	**primary**	**secondary**	**tertiary**
1x10^3 cells	4/4	6/6	6/6
1x10^2 cells	2/6	4/6	5/6

Supplementary Figure 1: Characterization of G7 renal CSCs. (A) Representative FACS analysis of G7 renal CSCs showing the expression of the mesenchymal stem cell markers CD105, CD73 and the embryonic stem cell marker SSEA4, but not of CD133, CD24 and HPCAM. **(B)** Representative micrograph showing the ability of G7 renal CSCs to form spheres when cultured in appropriate culture conditions. **(C)** Representative hematoxylin/eosin staining of tumors generated by G7 renal CSCs recovered from SCID mice. **(D)** Table representing the generation of serially transplantable tumors from renal CSCs.

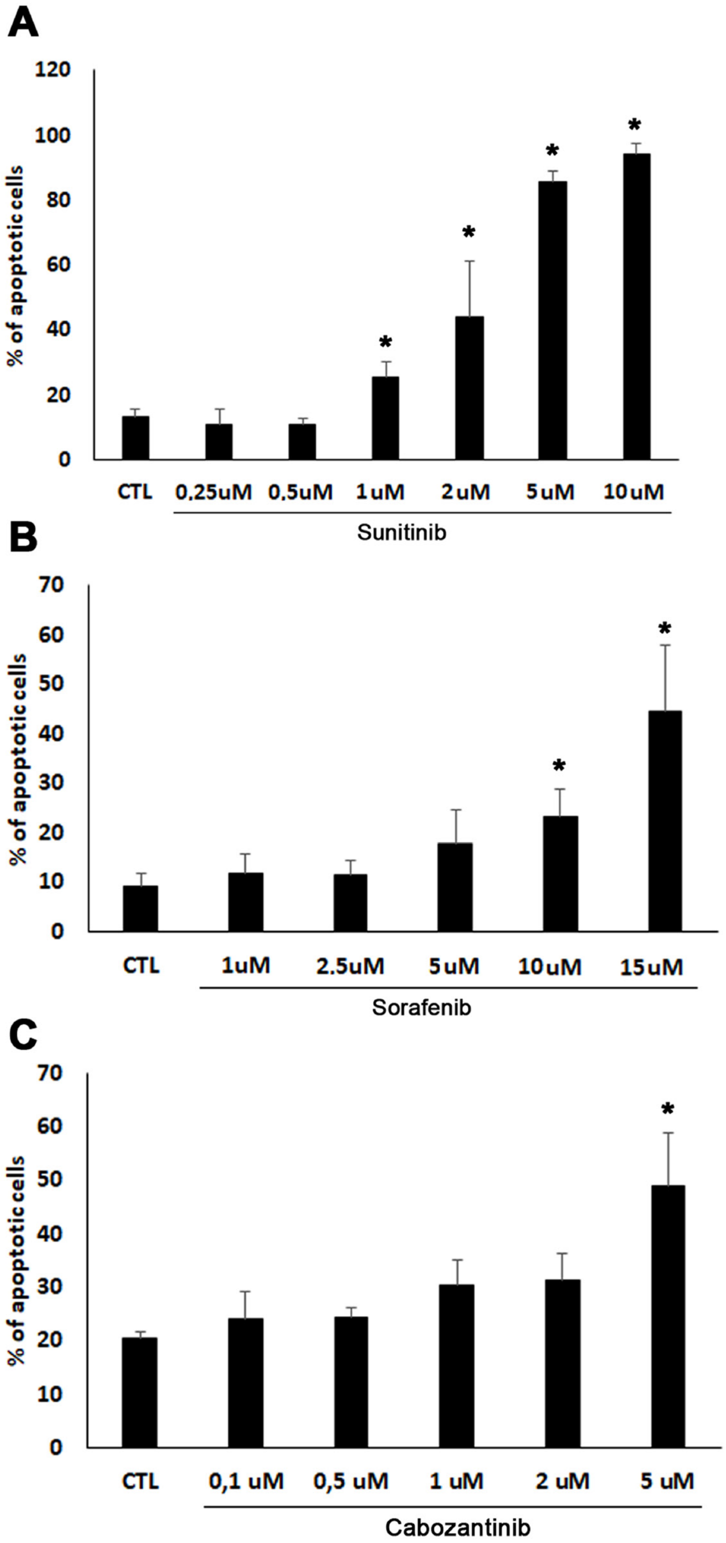

Supplementary Figure 2: TKIs dose/response on renal CSCs. Apoptosis analysis of G7 renal CSCs treated with different doses of **(A)** Sunitinib, **(B)** Sorafenib, or **(C)** Cabozantinib for 48 hours Sunitinib, Sorafenib, or Cabozantinib for 48 hours. Data are mean ± SD of three different experiments. Student's *t* test was performed: *= $p < 0.05$ vs CTL.
